# Two-color in-resin CLEM of Epon-embedded cells using osmium resistant green and red fluorescent proteins

**DOI:** 10.1038/s41598-020-78879-x

**Published:** 2020-12-14

**Authors:** Isei Tanida, Yoko Furuta, Junji Yamaguchi, Soichiro Kakuta, Juan Alejandro Oliva Trejo, Yasuo Uchiyama

**Affiliations:** 1 Department of Cellular and Molecular Neuropathology, Juntendo University Graduate School of Medicine, Tokyo, Japan; 2 Laboratory of Morphology and Image Analysis, Research Support Center, Juntendo University Graduate School of Medicine, Tokyo, Japan

**Keywords:** Imaging, Fluorescence imaging

## Abstract

In-resin CLEM of Epon embedded samples can greatly simplify the correlation of fluorescent images with electron micrographs. The usefulness of this technique is limited at present by the low number of fluorescent proteins that resist CLEM processing. Additionally, no study has reported the possibility of two-color in-resin CLEM of Epon embedded cells. In this study, we screened for monomeric green and red fluorescent proteins that resist CLEM processing. We identified mWasabi, CoGFP variant 0, and mCherry2; two green and one red fluorescent proteins as alternatives for in-resin CLEM. We expressed mitochondria-localized mCherry2 and histone H2B tagged with CoGFP variant 0 in cells. Green and red fluorescence was detected in 100 nm-thin sections of the Epon-embedded cells. In the same thin sections, we correlated the fluorescent signals to mitochondria and the nucleus using a scanning electron microscope. Similar results were obtained when endoplasmic reticulum-localized mCherry2 and histone H2B tagged with CoGFP variant 0 were expressed in the cells. Two-color in-resin CLEM of two cytoplasmic organelles, mitochondria and endoplasmic reticulum, was also achieved using mitochondria-localized mCherry2 and endoplasmic reticulum-localized mWasabi. In summary, we report three new fluorescent protein-alternatives suitable for in-resin CLEM of Epon-embedded samples, and achieved Epon-based two-color in-resin CLEM.

## Introduction

Fluorescence microscopy with multi-color fluorescent proteins is an essential tool in the field of cell biology. Using this technique we can visualize the intracellular localization of a target protein, cell-to-cell interactions, and the intracellular co-localization of two proteins^1^. A limitation of fluorescence microscopy is that it is difficult to analyze the cell ultrastructure. On the other hand, electron microscopy is suitable for analyzing ultrastructures. Correlative light-electron microscopy (CLEM) combines the advantage of fluorescent and electron microscopy and enables the analysis of fluorescent probe expression at the ultrastructural level^2^.

In classical CLEM, fluorescent images of cells are obtained after chemical fixation with paraformaldehyde and/or glutaraldehyde. After the acquisition of fluorescent images, the cells are treated with osmium tetroxide, dehydrated with ethanol, and embedded in epoxy resins. Electron microscopic images of cells in thin sections are obtained after sectioning the Epon-embedded specimens using an ultramicrotome with a diamond knife. As a detriment, the chemical modifications caused by fixation and the physical sectioning distort the sample’s morphology.

One of the most efficient ways to get the highest accuracy overlay of the fluorescent and electron microscopic imaging modalities during CLEM processing is to merge fluorescent and electron microscopic images from the same section following embedding in epoxy (or methacrylate) resins^3–5^. Epon-embedding is one of most robust and popular methods, because it preserves the ultrastructure of cells better and with higher contrast than other methacrylate-based resins (ex. GMA, LR white and Lowicryl resins). A disadvantage of this technique is that the fluorescent intensity of most fluorescent proteins is significantly weakened during CLEM processing. A proposed solution for these problems is to use Lowicryl HM20 (acrylate- and methacrylate-based) resins instead of Epon resins and high pressure freezing and freeze substitution techniques for in-resin CLEM with standard fluorescent proteins (mEGFP, mVenus, mRuby2, and YFP). However, these techniques require special instruments and special resins^3–6^.

Recently, it has been reported that two monomeric fluorescent proteins, mKate2 tagged with seven amino acids (GGGGSGL) (mKate2-GGGGSGL) and mEosEM retain their fluorescence after osmium staining and Epon embedding^7,8^. mKate2 is a far-red fluorescent protein^9^. mEosEM is a photoconvertible fluorescent protein^8^. The discovery of these proteins suggests that there may be other fluorescent proteins suitable for in-resin CLEM of Epon-embedded samples.

Considering the advantage of fluorescent proteins and Epon based in-resin CLEM in cell biology, multi-color in-resin CLEM of Epon-embedded cells should be developed. In the present study, we found monomeric green and red fluorescent proteins that retain their fluorescence after Epon-embedding. These proteins can be visualized using a fluorescence microscope with standard filter sets for green (excitation: 450–490 nm, dichroic mirror: 495 nm (LP), emission: 500–550 nm) and red (excitation: 540–580 nm, dichroic mirror: 585 nm (LP), emission: 592.5–667.5 nm) fluorescent probes. We also report on the achievement of two-color in-resin CLEM of Epon embedded cells using these proteins.

## Results

### Monomeric green and red fluorescent proteins retained their fluorescence after osmium tetroxide staining following paraformaldehyde-glutaraldehyde fixation

**Figure 1:**
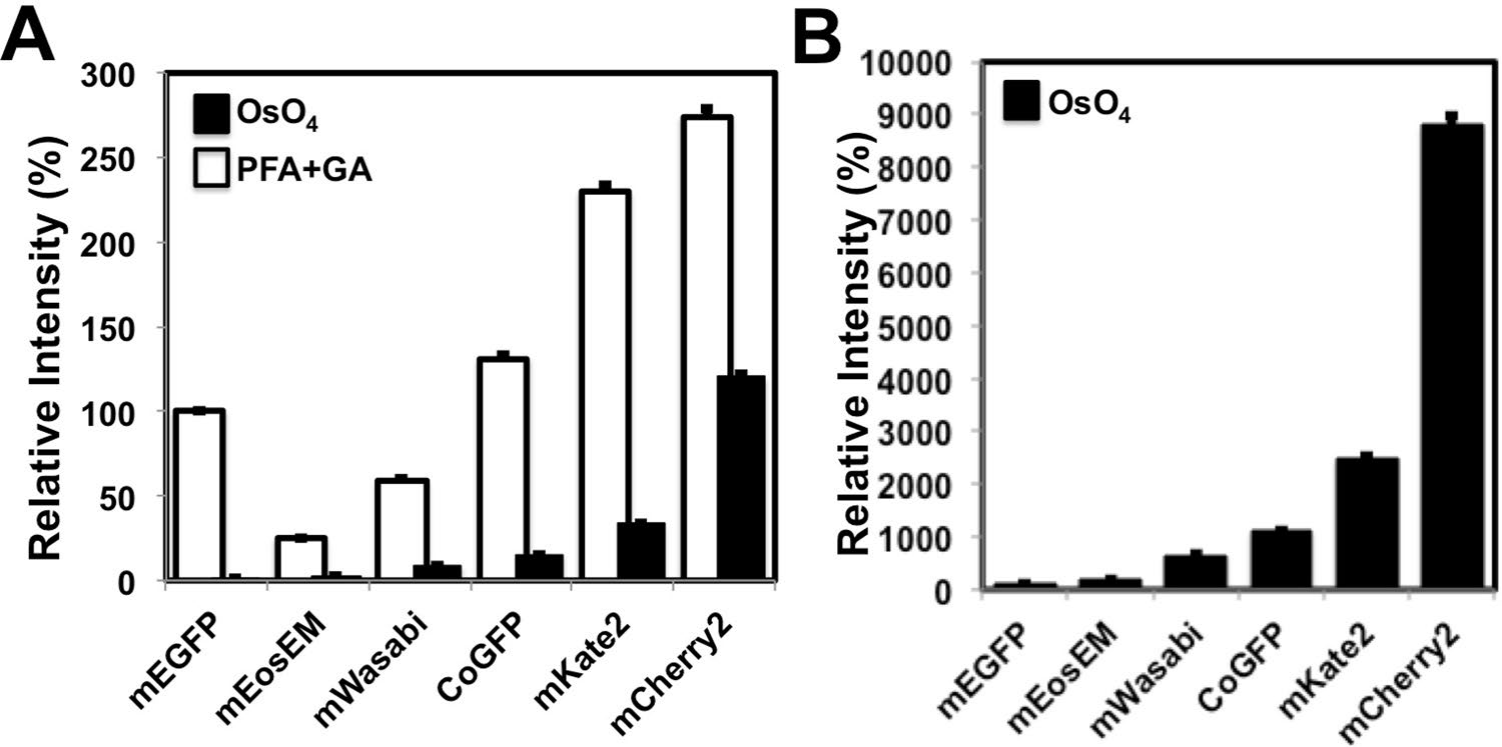
Fluorescent intensities of fluorescent proteins in the cells were quantified after the fixation with paraformaldehyde and glutaraldehyde and osmification. Cells expressing mEGFP, mWasabi, CoGFP, mEosEM, mCherry2, and mKate2 were fixed with a mixture of 2% paraformaldehyde and 2.5% glutaraldehyde at 4 °C for 1 h. After washing the cells in a HB solution three times, fluorescence images were obtained with a BZ-X810 fluorescence microscope (CCD monochrome camera, NIKON CFI plan Apochromat 40 × lens, gain + 16 dB) using filter sets for green (for mEGFP, mWasabi, CoGFP, and mEosEM) and red (for mCherry2 and mKate2) fluorescent probes (**PFA + GA,** white squares). Next, the fixed cells were treated with 1% osmium tetroxide at 4 °C for 10 min, washed with in HB solution three times, and incubated in TUK solution for multicolor at 4 °C for 10 min (**OsO**_**4**_, black squares). After washing the fixed cells with HB solution three times, fluorescence images were obtained with a BZ-X810 fluorescence microscope under the same conditions as described above. Fluorescent intensities of the cells (n > 340 in each fluorescent protein) were evaluated using imageJ software. (**A**) Relative intensity of each fluorescent protein was shown when fluorescent intensity of mEGFP after fixation with a mixture of paraformaldehyde and glutaraldehyde was regarded as 100%. (**B**) Relative intensity of each fluorescent protein was shown when fluorescent intensity of mEGFP after osmium-staining was regarded as 100%. Error bars indicate standard errors.

We first investigated whether or not monomeric green fluorescent proteins including mEGFP (monomeric EGFP with the A206K mutation) (λex max = 488 nm, λem max = 507 nm, pKa = 6.0)^10^, mWasabi (λex max = 493 nm, λem max = 509 nm, pKa = 6.5)^11^, mEosEM^8^, and CoGFP variant 0 isolated from *Cavernularia obesa* (CoGFPv0) (λex max = 498 nm, λem max = 507 nm, pKa = 6.0)^12^ retain their fluorescence after osmium-staining. Cells expressing each fluorescent protein were fixed with a mixture of 2% paraformaldehyde and 2.5% glutaraldehyde at 4 °C for 1 h, and stained with 1% osmium tetroxide at 4 °C for 10 min. The retained fluorescence of each green fluorescent protein was detected using a fluorescence microscope with a filter set for green fluorescent probe (Fig. 1 and Supplementary Fig. 1). After osmium-staining, fluorescent intensities of mEGFP, mEosEM, mWasabi, and CoGFP were decreased to respective 1.36 ± 0.02%, 11.10 ± 0.17%, 15.17 ± 0.28%, and 11.62 ± 0.18% (average ± SE) (Fig. 1A). CoGFPv0 and mWasabi retained brighter fluorescence than that of mEGFP under these conditions (Fig. 1B).

Next, we analyzed monomeric red fluorescent proteins: mCherry2 (λex max = 589 nm, λem max = 610 nm, pKa = 3.3)^13^ and mKate2 (λex max = 588 nm, λem max = 633 nm, pKa = 5.4). mCherry2 is a variant of mCherry that features higher brightness. In a previous study we showed that mKate2 retains its fluorescence after Epon-embedding^7^. The retained fluorescence of mCherry2 and mKate2 was compared (Fig. 1A,B). By osmium-staining, fluorescent intensities of mKate2 and mCherry2 were decreased to 14.75 ± 0.20%, and 43.88 ± 0.76% (average ± SE) respectively (Fig. 1A). mCherry2 retained brighter fluorescence than mKate2 under these conditions (Fig. 1B). Based on these results, we focused on mWasabi, CoGFPv0, and mCherry2 as candidates for two-color in-resin CLEM of Epon-embedded samples.

### mWasabi, CoGFP variant 0, and mCherry2 retained their fluorescence in thin sections of Epon embedded cells

We investigated whether mWasabi, CoGFPv0, and mCherry2 retain their fluorescence in 100 nm-thin sections of Epon embedded cells. Cells expressing mWasabi, CoGFPv0 and mCherry2 were fixed with a paraformaldehyde-glutaraldehyde mixture and stained with osmium tetroxide. Cells were then dehydrated with ethanol and finally embedded in Epon resins at 60 °C for 72 h. After preparation of 100 nm-thin sections from Epon-embedded samples, the fluorescence from each protein in the Epon-embedded cells was detected using a fluorescence microscope with filter sets for green (for mWasabi and CoGFP) or red (for mCherry2) fluorescent probes (Supplementary Fig. 2). The fluorescence from all proteins was detected well in the 100 nm-thin sections of Epon-embedded cells. In addition, the ultrastructures of Epon-embedded cells in the same section were observed using a scanning electron microscope. These results suggested that two-color in-resin CLEM of cells using these green and red fluorescent proteins can be achieved.

### Two color in-resin CLEM of nucleus, mitochondria and endoplasmic reticulum in the Epon-embedded cells was achieved using CoGFP variant 0 and mCherry2

**Figure 2:**
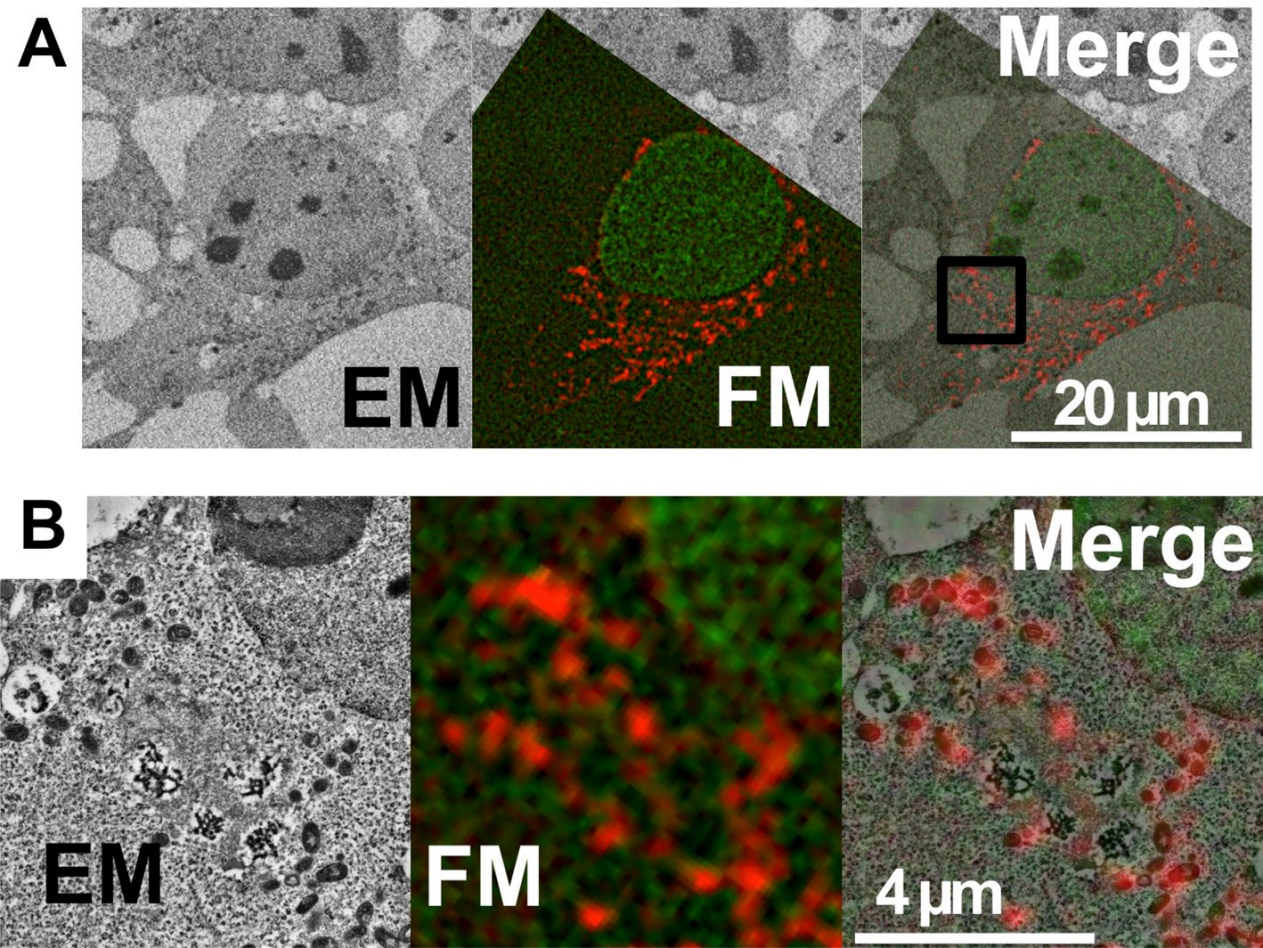
Two-color in-resin CLEM of nucleus and mitochondria was performed using H2B-CoGFPv0 and mCherry2-mito. Thin section (100 nm) of Epon-embedded cells expressing H2B-CoGFPv0 (green pseudo color) and mCherry2-mito (red pseudo color) was prepared. Fluorescent images (FM) were obtained in the presence of a TUK solution for multicolor with a BZ-X810 fluorescence microscope (CCD monochrome camera, NIKON CFI plan Apochromat 100 × Oil lens, gain + 16 dB, haze reduction) using filter sets for green and red fluorescent probes. Electron microscopic images (EM) were obtained with a Helios NanoLab 660 scanning electron microscope (a backscattered electron detector at a voltage of 2.0 kV with a current of 0.4 nA), and were processed with a method called “Contrast Limited Adaptive Histogram Equalization” using imageJ software with the plugin Enhance Local Contrast (CLAHE). The “Merge” is a merged image of the fluorescence image (FM) with an electron microscopic image (EM). The images in (**B**) indicate magnification of images corresponding to the boxed area in the Merge image in (**A**).

Using these green and red fluorescent proteins, we investigated whether two color in-resin CLEM of nucleus and mitochondria in the Epon-embedded cells could be achieved. For the localization of CoGFPv0 to the nucleus, we generated an expression plasmid of histone H2B-tagged with CoGFPv0 (H2B-CoGFPv0)^14^. For the localization of mCherry2 to mitochondria, we generated an expression plasmid of mCherry2-fused with a mitochondria-targeting signal of the ActA gene of *Listeria monocytogenes* (mCherry2-mito)^7,15^. To confirm the correct expression of the probes, we immunostained cells with lamin B1, a marker of nuclear membrane and TOM20, a mitochondrial marker. As expected, H2B-CoGFPv0 was detected inside lamin B1 and mCherry2-mito was co-localized with TOM20 (Supplementary Fig. 3). Cells expressing both proteins were fixed, stained and embedded using the same methodology as described in the previous section. After Epon-embedding, 100 nm thin sections were prepared. Analysis of the sections using a fluorescence microscope showed that the green fluorescence of H2B-CoGFPv0 was detected in the nucleus of cells with a filter set for green fluorescent probes (Fig. 2) and the red fluorescence of mCherry2-mito was detected with a filter set for red fluorescent probes. Electron microscopy analyses revealed that green fluorescent signals corresponded to the nucleus while the red fluorescent signals corresponded to the mitochondria.

**Figure 3:**
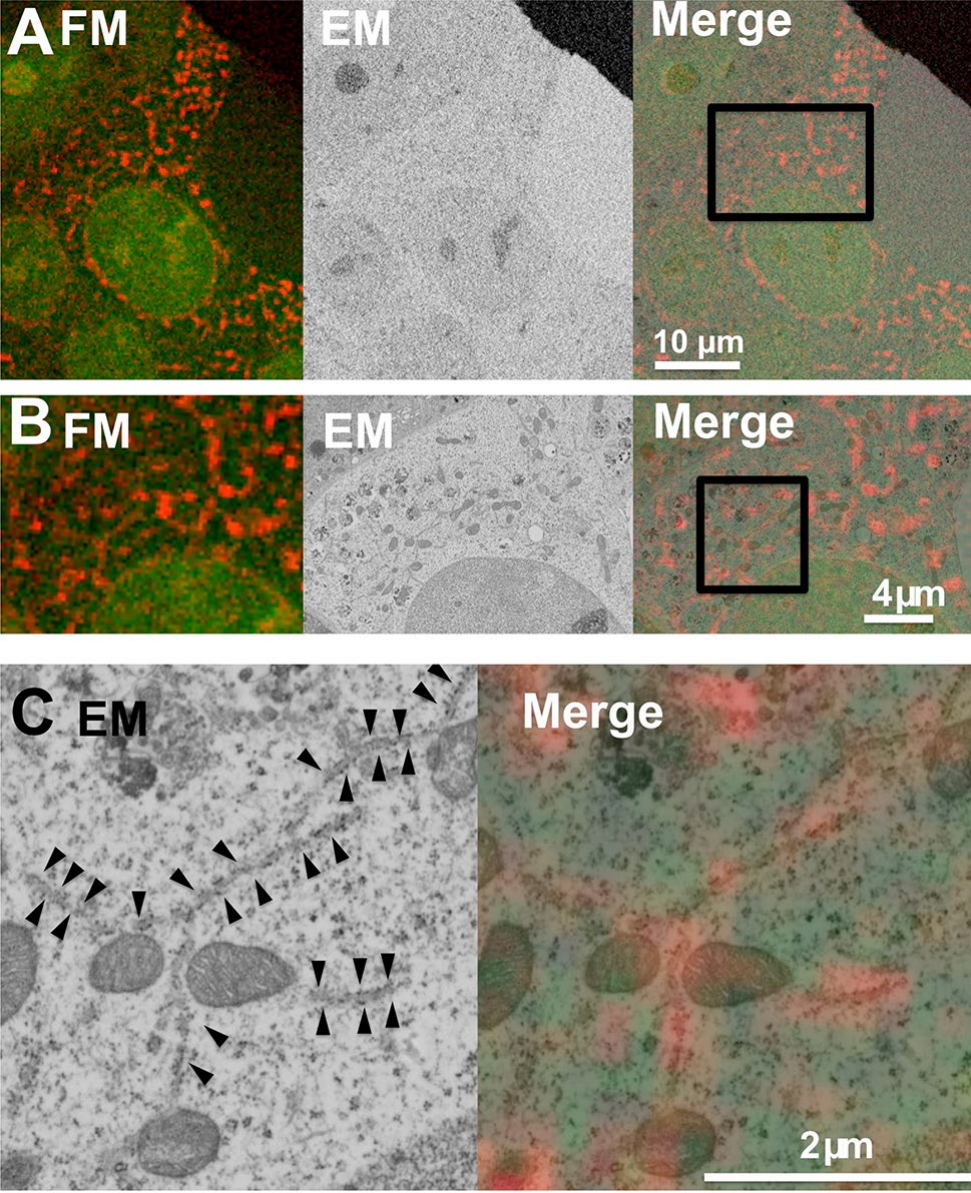
Two-color in-resin CLEM of nucleus and endoplasmic reticulum was performed using H2B-CoGFPv0 and mCherry2-ER. Thin section (100 nm) of Epon-embedded cells expressing H2B-CoGFPv0 (green pseudo color) and mCherry2-ER (red pseudo color) was prepared. Fluorescent images (FM) and electron microscopic images (EM) were obtained as described in Fig. 2. The “Merge” is a merged image of the fluorescence image (FM) with an electron microscopic image (EM). The images in (**B**) and (**C**) indicate magnification of images corresponding to the boxed area in the Merge image in respective (**A**) and (**B**). Arrowheads in (**C**) indicate red fluorescence-positive rough endoplasmic reticulum.

Next, we investigated whether two-color in-resin CLEM of nucleus and endoplasmic reticulum can be accomplished. For the localization of mCherry2 to endoplasmic reticulum, we generated an expression plasmid of mCherry2 fused with an ER-targeting sequence of calreticulin and the ER retrieval sequence, KDEL (mCherry2-ER). mCherry2-ER was co-localized with calnexin, a marker of endoplasmic reticulum (Supplementary Fig. 3). Cells expressing H2B-CoGFPv0 and mCherry2-ER were fixed, stained and embedded as described previously. In 100 nm-thin sections, we detected green and red fluorescence emitted from CoGFPv0 and mCherry2 (Fig. 3). In matching electron micrographs, we determined that the red fluorescent signals corresponded to the endoplasmic reticulum and the green fluorescent signals corresponded to the cell nucleus. These results showed that CoGFPv0 and mCherry2 are suitable for two-color in-resin CLEM of organelles in the Epon-embedded cells.

### Two color in-resin CLEM of endoplasmic reticulum and mitochondria in the Epon-embedded cells was achieved using mWasabi and mCherry2

**Figure 4:**
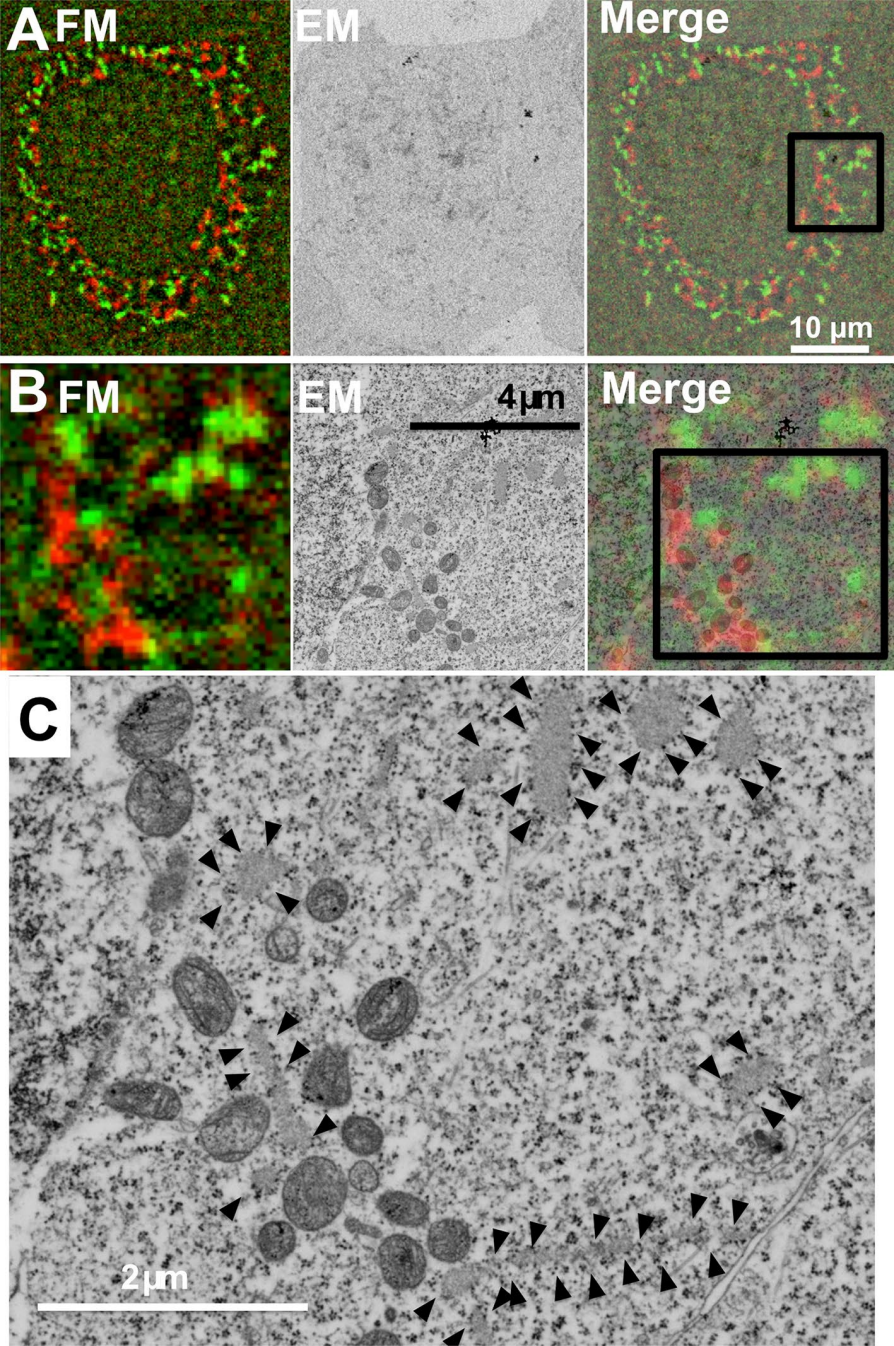
Two-color in-resin CLEM of nucleus and endoplasmic reticulum was performed using mWasabi-ER and mCherry2-mito. Thin section (100 nm) of Epon-embedded cells expressing mWasabi-ER (green pseudo color) and mCherry2-mito (red pseudo color) was prepared. Fluorescent images (FM) and electron microscopic images (EM) were obtained as described in Fig. 2. The “Merge” images in (**A**) and (**B**) are merged images of a fluorescence image of FM and EM. The images in (**B**) indicate magnification of images corresponding to the boxed area in the Merge image in (**A**). The EM images in (**C**) indicate magnification of image corresponding to the boxed area in the Merge image in (**B**). Arrowheads in (**C**) indicate green fluorescence-positive rough endoplasmic reticulum.

There is no report of a study using a green fluorescent protein targeting the endoplasmic reticulum for in-resin CLEM of Epon-embedded cells. We investigated the possibility of using two-color in-resin CLEM for analysis of cytoplasmic organelles: endoplasmic reticulum and mitochondria. We used mWasabi fused with an ER-targeting sequence of calreticulin and the ER retrieval sequence, KDEL (mWasabi-ER). In immunostained samples, mWasabi-ER co-localized with calnexin thereby confirming the correct expression of the construct (Supplementary Fig. 3). Fluorescence microscopy confirmed the detection of green and red fluorescence, of mWasabi-ER and mCherry2-mito respectively, in thin sections of Epon-embedded cells expressing both proteins (Fig. 4). Electron microscopy analyses of the same thin sections revealed that the red fluorescence from mCherry2-mito corresponded to the mitochondria, while green fluorescence from mWasabi-ER corresponded to the endoplasmic reticulum. These results showed that two-color in-resin CLEM of intracellular organelles can be achieved using these fluorescent proteins.

## Discussion

We discovered that two green fluorescent proteins, mWasabi and CoGFPv0, and one red fluorescent protein, mCherry2, can retain their fluorescence after osmium-staining. Under our experimental conditions, mWasabi and CoGFPv0 retain brighter fluorescence than mEosEM after osmium staining (Fig. 1B). mCherry2 retains brighter red fluorescence than mKate2-GGGGSGL under the same conditions. When the fluorescent intensities of these fluorescent proteins are compared before and after osmium-staining, these intensities of mEGFP, mEosEM, mWasabi, CoGFP, mKate2, and mCherry2 decrease to respective 1.36 ± 0.02%, 11.10 ± 0.17%, 15.18 ± 0.28%, 11.62 ± 0.18%, 14.75 ± 0.21%, and 43.86 ± 0.76% after osmium-staining (Fig. 1A). Considering fluorescent intensities of these fluorescent proteins (Fig. 1B) and autofluorescence of 100 nm-thin sections of epoxy resins (Supplementary Fig. 2), we focused on CoGFP and mWasabi as green fluorescent proteins, and mCherry2 as a red fluorescent protein. Using these proteins, we achieved two-color in-resin CLEM of cells and organelles in 100 nm-thin sections of the Epon-embedded samples.

Which is better as a green fluorescent protein suitable for in-resin CLEM CoGFPv0 or mWasabi? CoGFPv0 tends to be brighter than mWasabi after osmium-staining (Fig. 1). However, there is little information of CoGFPv0 fusion proteins for intracellular imaging using fluorescence microscopy, while mWasabi fusion proteins are available for intracellular imaging. We made expression vectors for CoGFPv0 fusion proteins for endoplasmic reticulum, the Golgi, and mitochondria, but these proteins tend to make aggregates and/or distort the normal morphology. It is better to employ mWasabi as a first candidate of a green fluorescent protein for in-resin CLEM of organelles in Epon-embedded specimens. For labeling cells, CoGFPv0 is a first candidate, since it is easier to detect green fluorescence than mWasabi in the thin sections of Epon embedded cells.

An unexpected finding of our study is that mCherry2, one of many mRFP variants, retains brighter fluorescence than mKate2 after osmium-staining (Fig. 1). This is because it has been reported that mApple, another variant of mRFP, retains less fluorescence than mKate2 after osmium staining^7^. Brightness of mApple at pH 7.0 is about 2.1 and 1.5 fold brighter than that of mCherry2 and mKate2^16^. Therefore, there is no answer as to why mCherry2, but not mApple, retains brighter red fluorescence than mKate2 after osmium staining.

Interestingly, mEGFP also retains faint weak fluorescence after osmium staining, while it requires about 100-fold longer exposure than that before osmium staining (Fig. 1). It could be possible to achieve in-resin CLEM of Epon-embedded specimen using mEGFP, if the sensitivity of fluorescence microscopy is improved significantly.

## Materials and methods

### Cells, media, and materials

HEK293 cells were obtained from the American Type Culture Collection and were cultured in Dulbecco’s Modified Eagle’s Medium (FUJIFILM Wako Chemicals, #045–30,285) containing 10% fetal calf serum (JRH Biosciences/Sigma-Aldrich, 12603C). FuGENE HD transfection reagent was used to introduce the plasmid into cells (Promega, E2311).

pmCherry2-C1 was a gift from Michael Davidson (Addgene plasmid # 54563; http://n2t.net/addgene:54563 ; RRID:Addgene_54563). For the expression of the CoGFPv0, mWasabi, and mCherry2 and their fusion proteins, we used pCAGGS^17^ and pAAV-CMV (TAKARA, 6673) plasmids. pmEosEM-C, an expression plasmid for mEosEM, was described previously^7^. To generate an expression plasmid for mWasabi and mCherry2, DNA fragments encoding these proteins was generated by a KOD one PCR master mix (TOYOBO, KMM-201) using respective pmWasabi-C (Allele Biotechnology, ABP-FP-WCNCS10) and pmCherry2-C1 as a template, and introduced into pCAGGS plasmid (respective pCAG-mWasabi-G and pCAG-mCherry2-G plasmids). To generate an expression plasmid for CoGFPv0 and mEGFP, DNA fragments encoding codon-optimized CoGFP variant 0 (GenBank Ac. No. LC576825) and mEGFP (GenBank Ac. No. LC384836) were synthesized by Integrated DNA Technologies and introduced into pCAGGS plasmid (pCAG-CoGFP-G and pCAG-mEGFP-HO-G plasmids).

For the expression of H2B-CoGFPv0 under the control of the CMV promoter, a DNA fragment encoding histone H2B was generated by a KOD one PCR master mix using primer sets (H2B-cagarm-Nhe-ATG-F: GTG CTG TCT CAT CAT TTT GGC AAA GCT AGC GCC ACC ATG CCA GAG CCA GCG AAG TCT GCT CCC GCC, H2B-CGr1-atgarm-Rv: GTT CTC TGG AAT GGA CAT CTT AGC GCT GGT GTA CTT GGT GAT GGC CTT) and genomic DNA isolated from HEK293 cells as a template. DNA fragments encoding histone H2B and CoGFPv0 were introduced into pAAV-CMV vector (pAV-H2B-CoGFP). For the expression of mCherry2-mito under the control of the CAG promoter, a DNA fragment encoding mCherry2-fused with a mitochondria-targeting signal of the ActA gene of *Listeria monocytogenes* was introduced into pCAG (pCAG-mCherry2-mito). For the expression of the mWasabi-ER and mCherry2-ER under the control of the CAG promoter, DNA fragments encoding mWasabi and mCherry2 fused with an ER-targeting sequence of calreticulin and the ER retrieval sequence, KDEL^18^, was introduced into pCAG plasmid (pCAG-mWasabi-ER and pCAG-mCherry2-ER)^19,20^.

### Sample-preparation, fluorescent microscopy, and electron microscopy for in-resin CLEM

Cells expressing fluorescent proteins and their fusion proteins were prefixed with a fixation solution containing 2% paraformaldehyde and 2.5% glutaraldehyde at 4 °C for 1 h^21^. The fixed cells were washed three times with a HB solution (FUJIFILM WAKO Chemicals, # 080-10591). Fixed cells were post-fixed in 1% osmium tetroxide at 4 °C for 10 min, and washed three times with a HB solution, incubated in TUK solution for multicolor (FUJIFILM WAKO Chemicals, # 208-21161) at 4 °C for 10 min to recover the fluorescence of the fluorescent proteins, and were washed three times with a HB solution. Cells were dehydrated with a graded series of ethanol, and embedded in Epon812 (Oken shoji) at 60 °C for 72 h. Thin sections (100 nm) were cut with an ultramicrotome UC6 (Leica) and placed on glass cover slips that were coated with Pt/Au using an ion sputter E-1010 (Hitachi). Sections were observed in a TUK solution for multicolor using a BZ-X810 fluorescence microscope (Keyence) with filter sets for green (excitation: 450–490 nm, dichroic mirror: 495 nm (LP), emission: 500–550 nm) (Keyence, # OP-87763), red (excitation: 540–580 nm, dichroic mirror: 585 nm (LP), emission: 592.5–667.5 nm) (Keyence, # OP-87765), and blue (excitation: 340–380 nm, dichroic mirror: 400 nm (LP), emission: 415–485 nm) (Keyence, # OP-87762) fluorescent probes. Fluorescence of fluorescent proteins in the cells of Epon-embedded samples was observed in the TUK solution. After observation, the thin sections were washed with distilled water, dried overnight at room temperature, stained with uranyl acetate and lead citrate, and observed via SEM (Helios NanoLab 660, FEI). The SEM images were obtained using a backscattered electron detector (CBS detector) at a voltage of 2.0 kV with a current of 0.4 nA as described previously^7^. Electron microscopic images were processed with a method “Contrast Limited Adaptive Histogram Equalization” using an imageJ software with a plugin Enhance Local Contrast (CLAHE)^22^ when indicated.

## Supplementary Information

Supplementary Information

## Data Availability

The DNA sequences encoding the ORFs used in this study were listed in Supplemental data.

## Abbreviations

CLEM: Correlative light-electron microscopy
ER: Endoplasmic reticulum
SEM: Scanning electron microscopy
