# Supplementary material for: Two-color in-resin CLEM of Epon-embedded cells using osmium resistant green and red fluorescent proteins
Source: Sci Rep. 2020 Dec 14;10:21871. doi: 10.1038/s41598-020-78879-x (PMC7736269; doi:10.1038/s41598-020-78879-x)
Supplement: Supplementary file 1 — Supplementary Information [file 41598_2020_78879_MOESM1_ESM.docx]

**Two-color in-resin CLEM of Epon-embedded cells using osmium resistant green and red fluorescent proteins**

Isei Tanida^1*^, Yoko Furuta^1^, Junji Yamaguchi^1,2^, Soichiro Kakuta^1,2^, Juan Alejandro Oliva Trejo^1^, and Yasuo Uchiyama^1*^

^1^ Department of Cellular and Molecular Neuropathology, Juntendo University Graduate School of Medicine, Tokyo, Japan

^2^ Laboratory of Morphology and Image Analysis, Research Support Center, Juntendo University Graduate School of Medicine, Tokyo, Japan

***** Correspondence: tanida@juntendo.ac.jp (I.T.) and y-uchi@juntendo.ac.jp (Y.U.); Tel: +81-3-3813-3111 ex 3601 (I.T.)

**Supplementary Fig. 1. Several fluorescent proteins retain their fluorescence after osmium tetroxide staining following the fixation with a mixture of paraformaldehyde and glutaraldehyde.** HEK293 cells expressing each fluorescent protein were fixed with a mixture of paraformaldehyde and glutaraldehyde at 4ºC for 1 h. Fluorescent images (**PFA+GA**) were obtained with a BZ-X810 fluorescence microscope (Keyence) (CCD monochrome camera, NIKON CFI60 series 20x lens, gain +16 dB) using GFP (for **mEGFP**, **mWasabi**, **CoGFPv0**, and **mEosEM**; green pseudo color) or Texas Red filter sets (for **mCherry2** and **mKate2**-GGGGSGL; red pseudo color). Thereafter, cells were treated with 1% osmium tetroxide at 4 ºC for 10 min. Fluorescent images (**OsO4**) were obtained with a BZ-X810 fluorescence microscope (CCD monochrome camera, NIKON CFI60 series 20x lens, gain +16 dB) using GFP or Texas Red filter sets. Exposure times were indicated at the bottom of each fluorescent image. Scale bars, 20 µm.
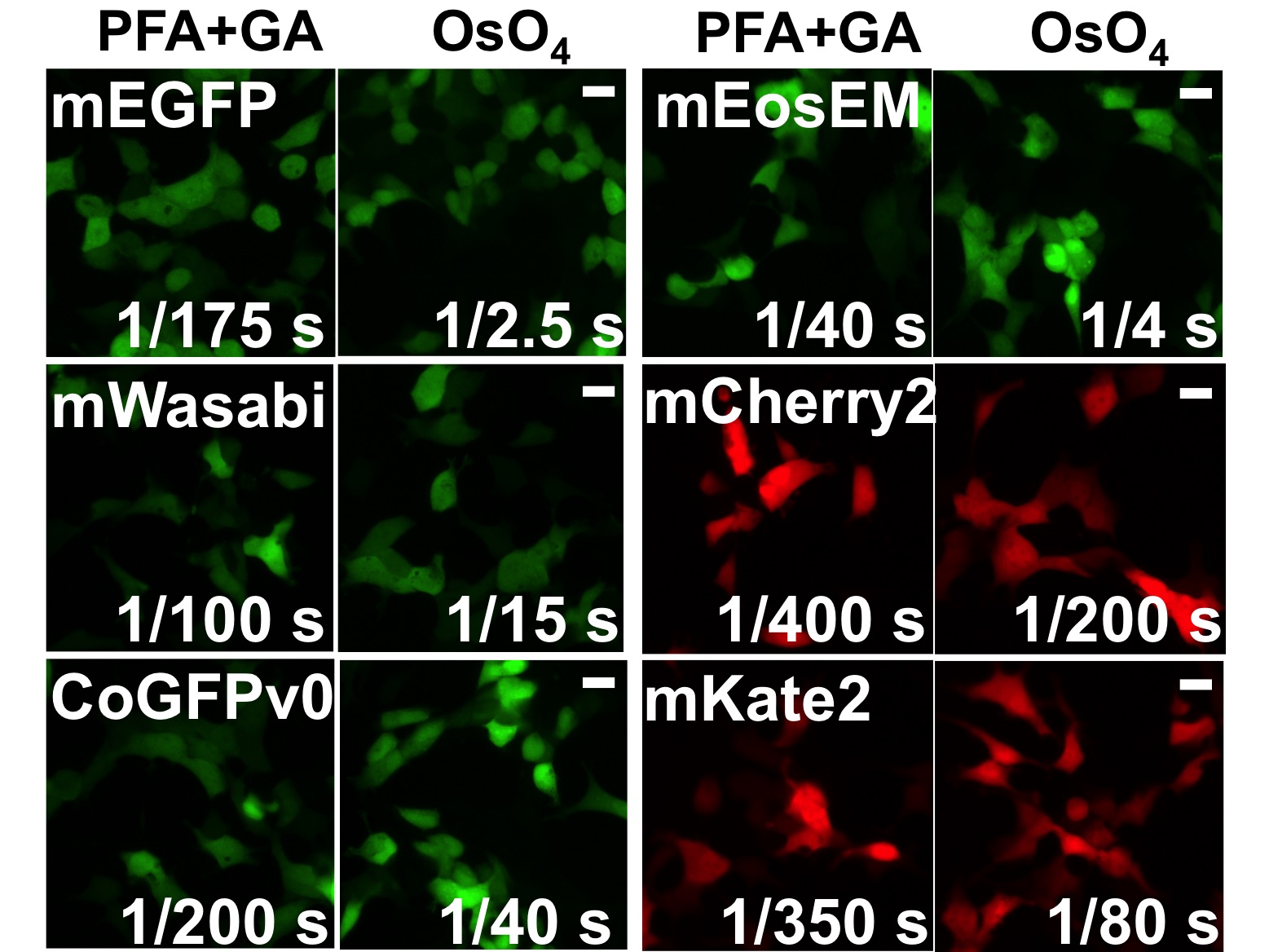


**Supplementary Fig. 2. CoGFP variant 0, mWasabi, and mCherry2 retain their fluorescence in thin sections of Epon-embedded cells.** Cells expressing mWasabi (**A-D**), CoGFPv0 (**E-H**), and mCherry2 (**I-L**) were fixed with a mixture of paraformaldehyde and glutaraldehyde, stained with osmium tetroxide, dehydrated with a series of ethanol, and embedded in Epon resins. After preparation of thin sections (100 nm), fluorescent images (panels **2-4** in **A, E, and I**) were obtained with a BZ-X810 fluorescence microscope (CCD monochrome camera, NIKON CFI plan Apochromat 10x lens, gain +8 dB, 2x2 binning) using filter sets for green (**2**; green pseudo color), red (**3**; red pseudo color), and blue fluorescent probes (**4**; blue pseudo color). The images in panel **1** are the merged images of the phase contrast and the three fluorescent images of panels 2-4. The fluorescent images in **B**, **F and J** are the magnified images of the area corresponding to the white squares in respective **A-2**, **E-2 and I-3** obtained with a BZ-X810 fluorescence microscope (CCD monochrome camera, NIKON CFI plan Apochromat 40x lens, gain +8 dB). Electron microscopic images (**C, D, G, H, K,** and **L**) were obtained with a Helios NanoLab 660 scanning electron microscope (a backscattered electron detector at a voltage of 2.0 kV with a current of 0.4 nA). The scanned SEM images in **C**, **G**, and **K** are the images of the area corresponding to the white squares in respective **B**, **F**, and **J**. The EM images in **D**, **H**, and **L** are images of the area corresponding to the black squares in respective **C**, **G**, and **K**.


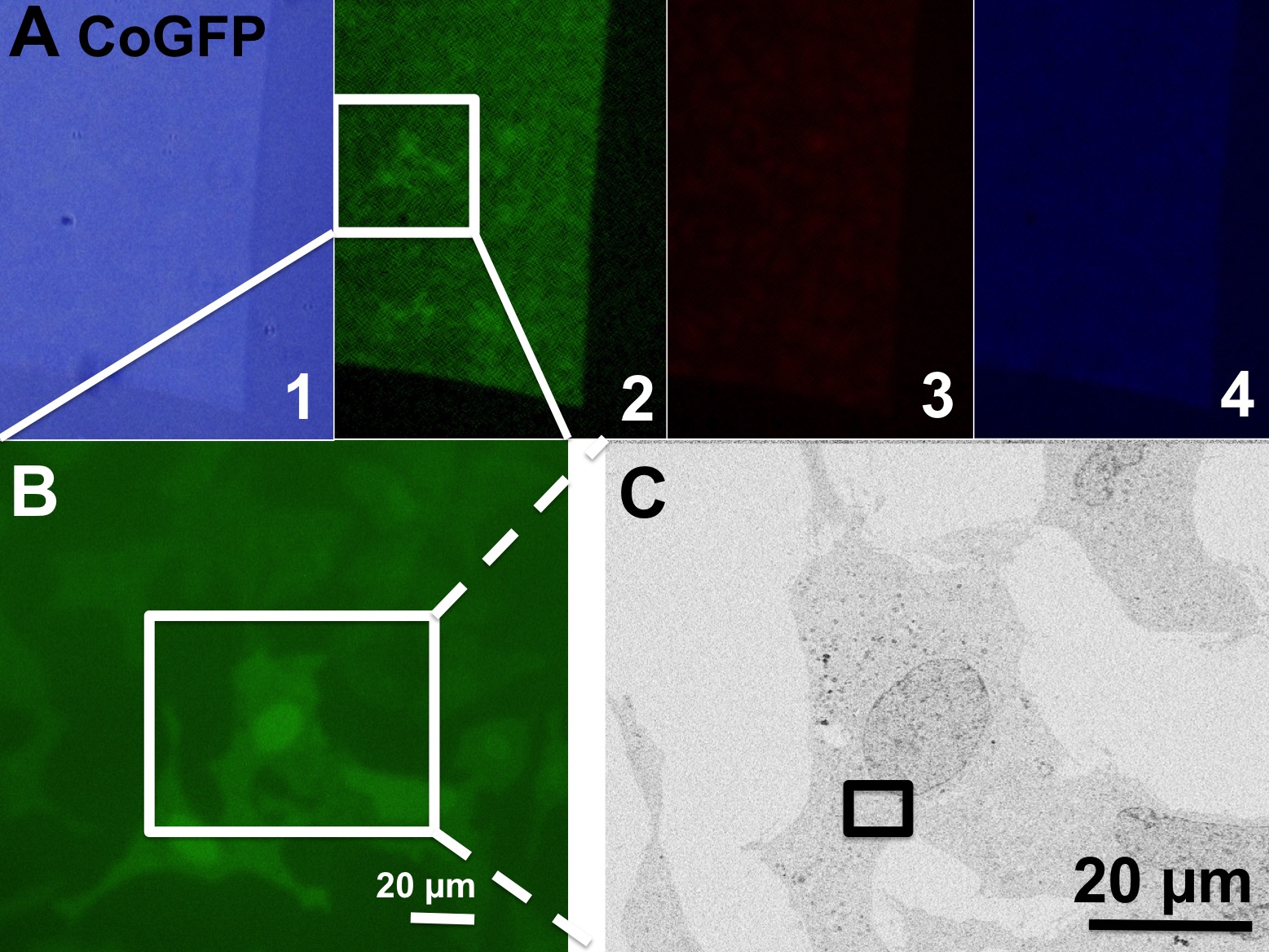

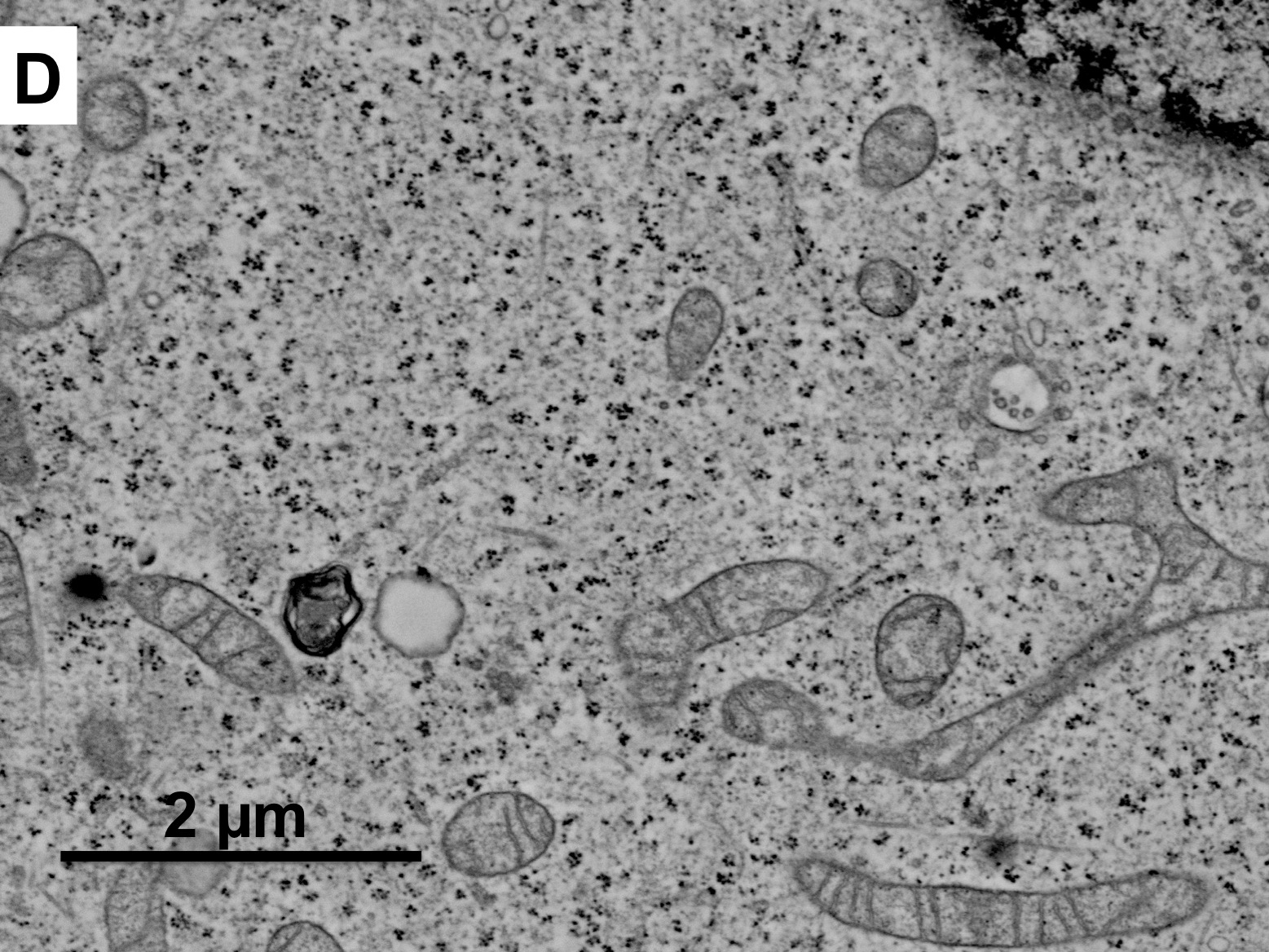

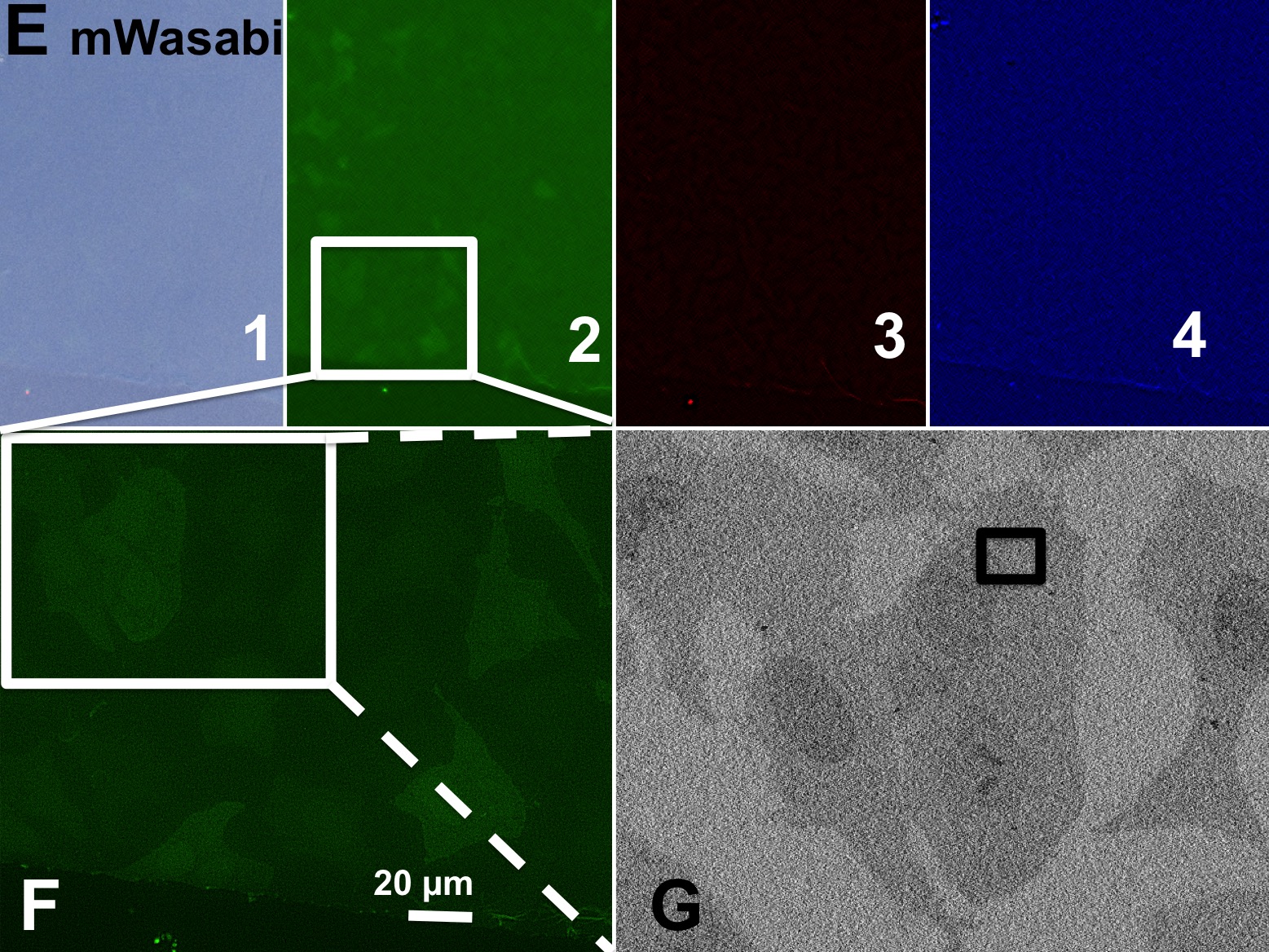

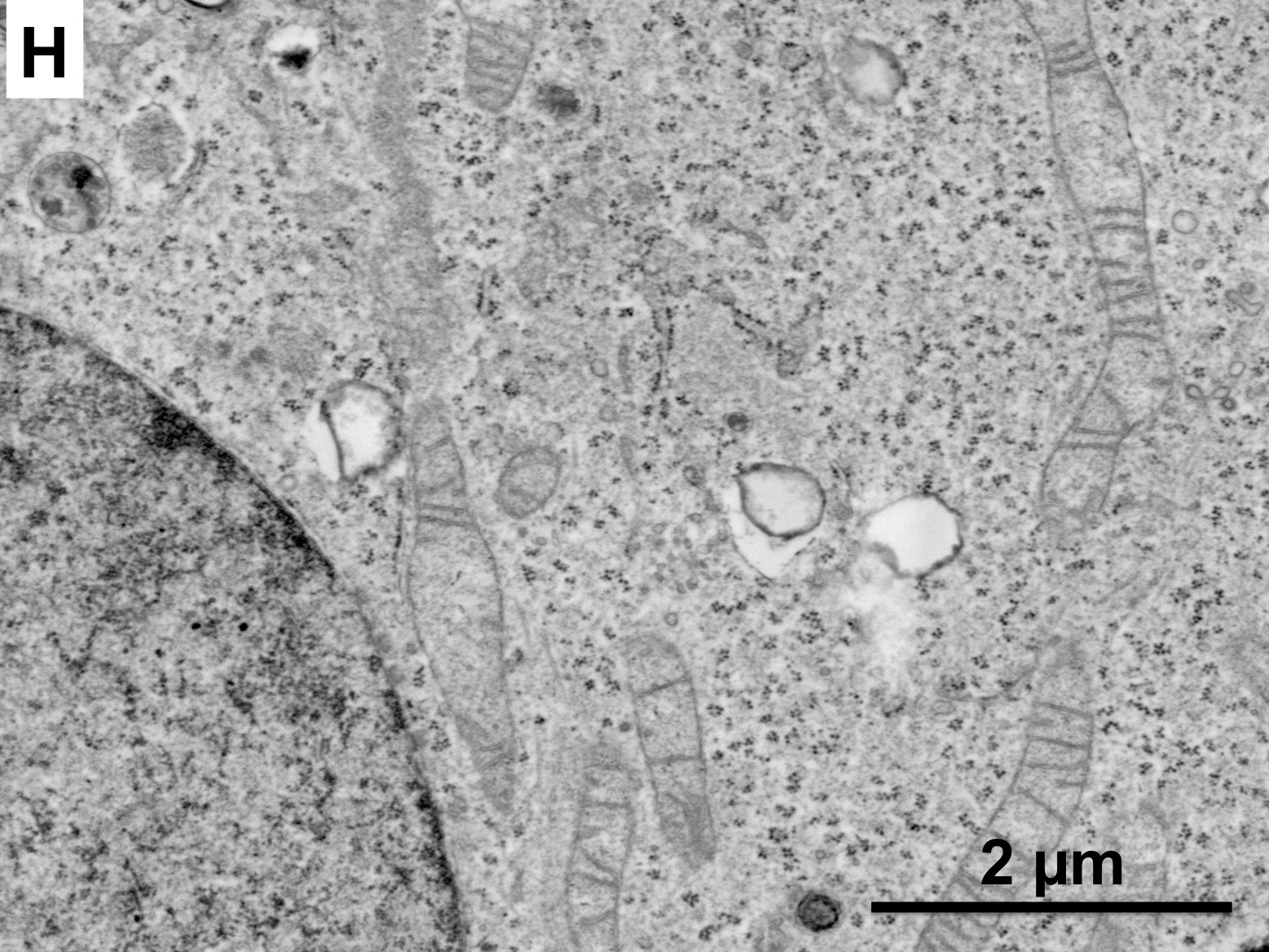

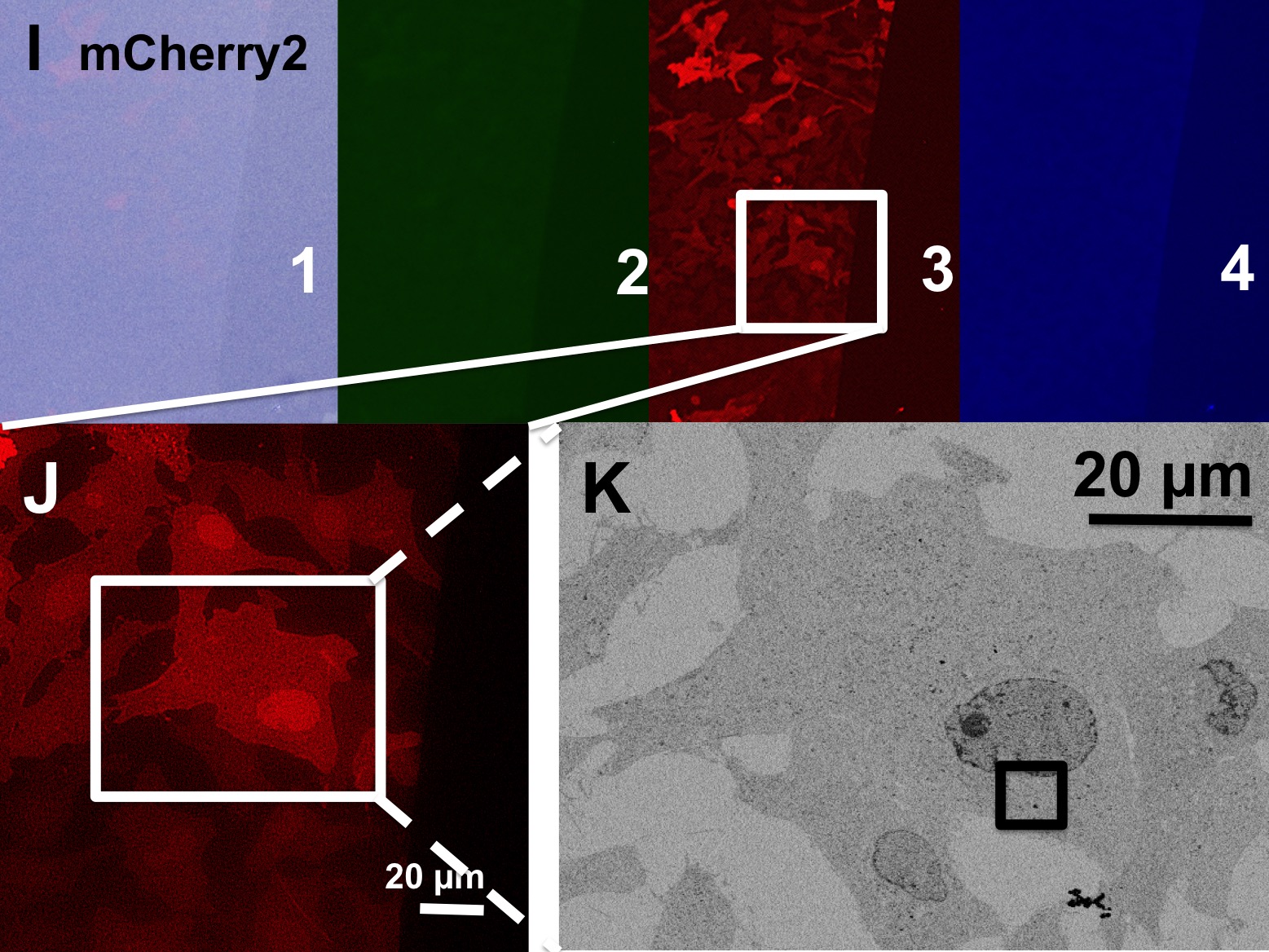


**
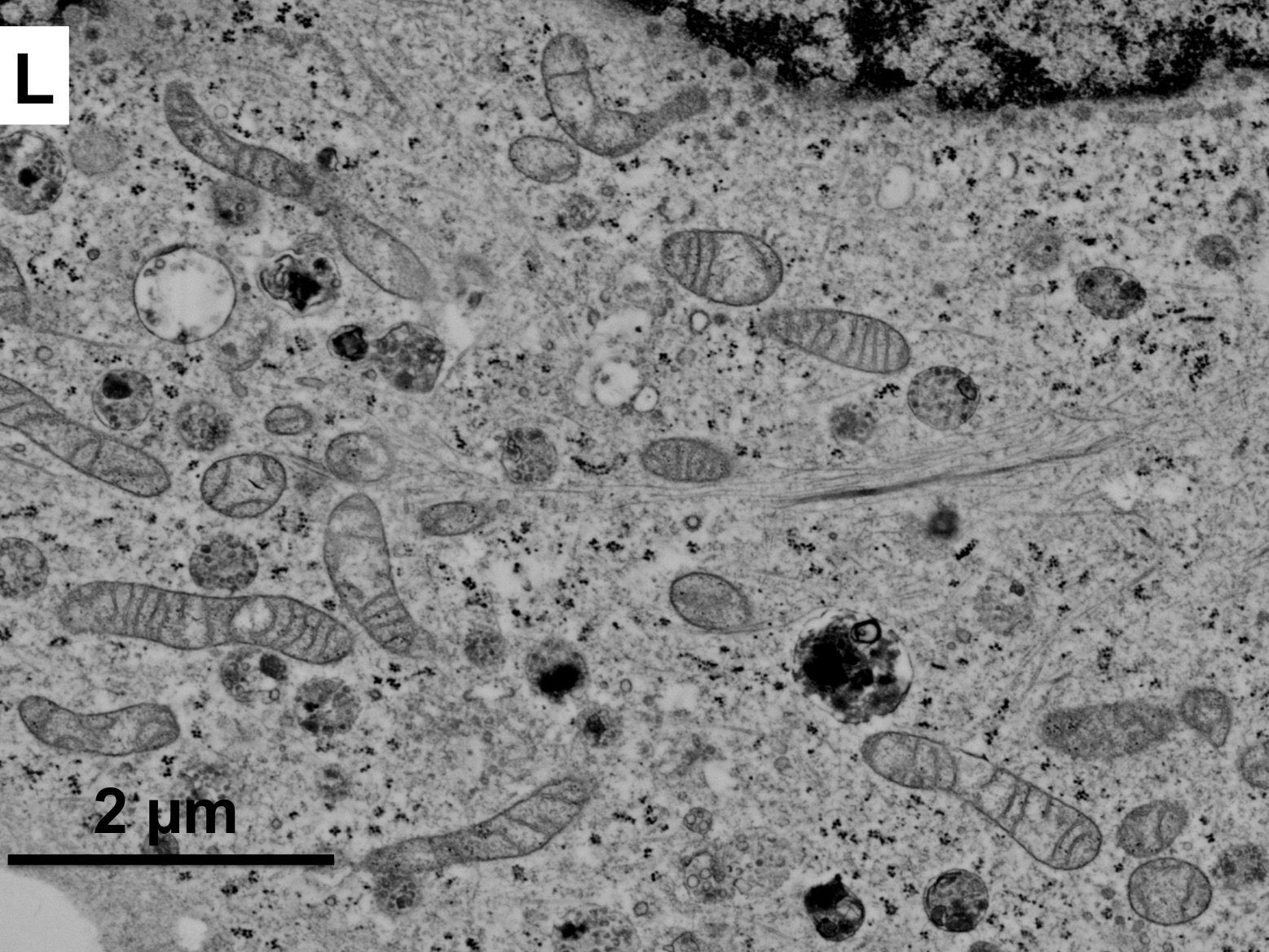
**

**Supplementary Fig. 3. H2B-CoGFP, mWasabi-ER, mCherry2-mito, and mCherry2-ER proteins localize to the respective targetted organelles.** The H2B-CoGFPv0 (**A**, **H2B-CoG**), mCherry2-mito (**B**, **mC2-mt**), mWasabi-ER (**C**, **mW-ER**), and mCherry2-ER (**D**, **mC2-ER**) proteins were expressed in HEK293 cells. The cells were fixed with 4% paraformaldehyde, and permeabilized with phosphate buffered serum containing 0.05% digitonin. After blocking the cells in phosphate buffered serum containing 1% BSA, rabbit polyclonal anti-**lamin B1** (nuclear membrane, ProteinTech, #12987-1-AP, x100 dilution), anti-**calnexin** (endoplasmic reticulum, Abcam, # ab22595, x100 dilution), and anti-**TOM20** antibodies (mitochondria, Abcam, #ab78547, x1000 dilution) were incubated in a phosphate buffered serum containing 0.1% BSA. As a seconday atibody, Alexa Fluor 647-conjugated goat anti-rabbit IgGs antobody (ThermoFisher/ Invitrogen, # A-21245) was employed. The fluorescent images were obtained with a BZ-X810 fluorescence microscope (Keyence) using GFP (green pseudo color), Texas Red (red pseudo color), and Cy5 (cyan pseudo color) filters (CCD monochrome camera, NIKON CFI plan Apochromat 100x Oil lens, gain +16 dB, haze reduction in **B**-**D**). Scale bars, 10 µm. The “**Merge**” is a merged image of the fluorescent image of the fluorescent protein (**left**) with Alexa Fluor 647-fluorescent image (**middle**) in each panel.

**
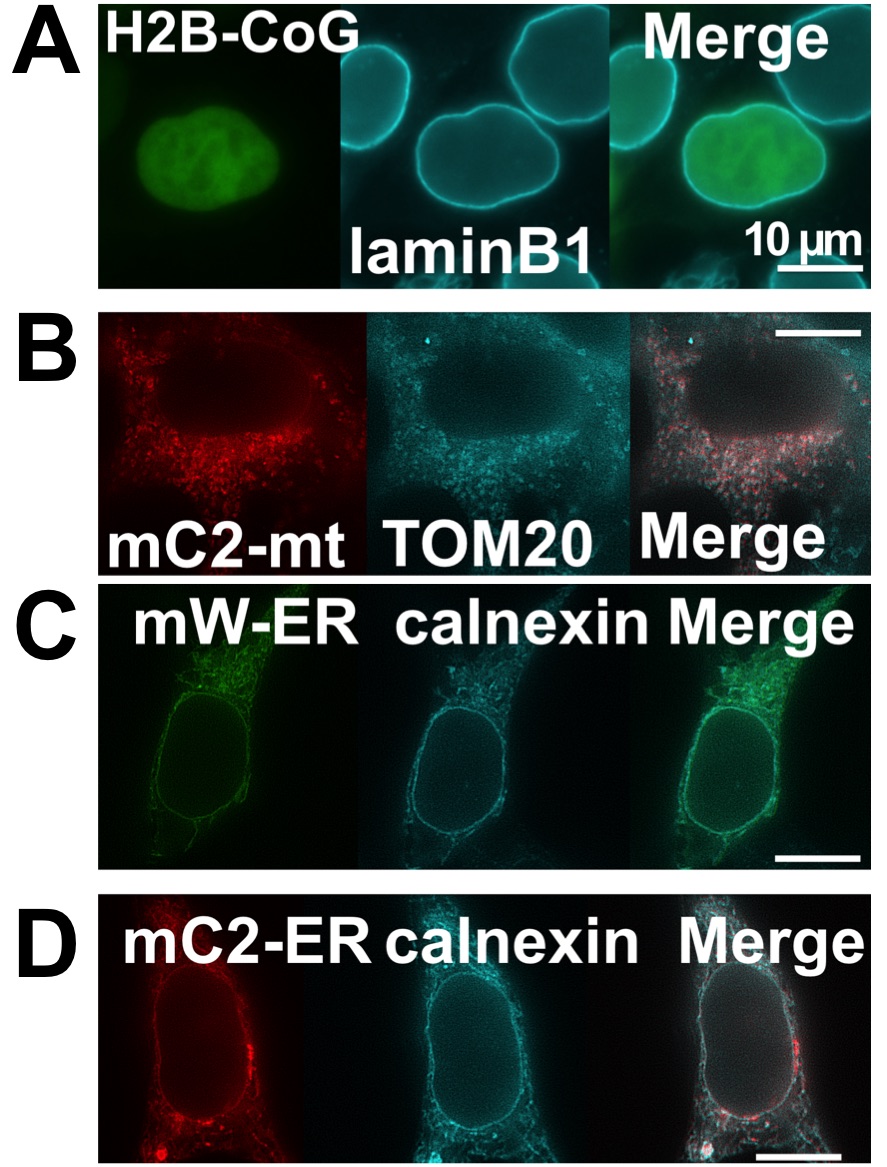
**

Supplementary Data. The DNA sequences encoding the ORFs used in this study.

| Humanized mEGFP | ATGGTGAGCAAGGGCGAGGAGCTGTTCACCGGCGTGGTGCCCATCCTGGTGGAGCTGGACGGCGACGTGAACGGCCACAAGTTCAGCGTGAGCGGCGAGGGCGAGGGCGACGCGACCTACGGCAAGCTGACCCTGAAGTTCATCTGCACCACCGGCAAGCTGCCCGTGCCCTGGCCCACCCTGGTGACCACCCTGACCTACGGCGTGCAGTGCTTCAGCCGCTACCCCGACCACATGAAGCAGCACGACTTCTTCAAGAGCGCCATGCCCGAGGGCTACGTGCAGGAGCGCACCATCTTCTTCAAGGACGACGGCAACTACAAGACCCGCGCCGAGGTGAAGTTCGAGGGCGACACCCTGGTGAACCGCATCGAGCTGAAGGGCATCGACTTCAAGGAGGACGGCAACATCCTGGGCCACAAGCTGGAGTACAACTACAACAGCCACAACGTGTACATCATGGCCGACAAGCAGAAGAACGGCATCAAGGTGAACTTCAAGATCCGCCACAACATCGAGGACGGCAGCGTGCAGCTGGCCGACCACTACCAGCAGAACACCCCCATCGGCGACGGCCCCGTGCTGCTGCCCGACAACCACTACCTGAGCACCCAGAGCAAGCTGAGCAAGGACCCCAACGAGAAGCGCGACCACATGGTGCTGCTGGAGTTCGTGACCGCCGCCGGCATCACCCTGGGCATGGACGAGCTGTACAAG |
| --- | --- |
| mWasabi | ATGGTGAGCAAGGGCGAGGAGACCACAATGGGCGTAATCAAGCCCGACATGAAGATCAAGCTGAAGATGGAGGGCAACGTGAATGGCCACGCCTTCGTGATCGAGGGCGAGGGCGAGGGCAAGCCCTACGACGGCACCAACACCATCAACCTGGAGGTGAAGGAGGGAGCCCCCCTGCCCTTCTCCTACGACATTCTGACCACCGCGTTCAGTTACGGCAACAGGGCCTTCACCAAGTACCCCGACGACATCCCCAACTACTTCAAGCAGTCCTTCCCCGAGGGCTACTCTTGGGAGCGCACCATGACCTTCGAGGACAAGGGCATCGTGAAGGTGAAGTCCGACATCTCCATGGAGGAGGACTCCTTCATCTACGAGATACACCTCAAGGGCGAGAACTTCCCCCCCAACGGCCCCGTGATGCAGAAGGAGACCACCGGCTGGGACGCCTCCACCGAGAGGATGTACGTGCGCGACGGCGTGCTGAAGGGCGACGTCAAGATGAAGCTGCTGCTGGAGGGCGGCGGCCACCACCGCGTTGACTTCAAGACCATCTACAGGGCCAAGAAGGCGGTGAAGCTGCCCGACTATCACTTTGTGGACCACCGCATCGAGATCCTGAACCACGACAAGGACTACAACAAGGTGACCGTTTACGAGATCGCCGTGGCCCGCAACTCCACCGACGGCATGGACGAGCTGTACAAG |
| Humanized CoGFP variant 0 | ATGTCCATTCCAGAGAACAGCGGCCTGACAGAGGAAATGCCGGCCCAGATGAACCTGGAGGGGGTCGTAAATGGCCATGCGTTTTCAATGGAAGGGATCGGGGGTGGAAACATACTGACTGGTATTCAAAAGTTGGACATTCGAGTGATTGAGGGTGACCCGCTCCCTTTCTCTTTTGACATCCTCTCCGTGGCATTTCAGTATGGTAATAGAACGTACACTTCCTATCCAGCAAAGATACCTGACTATTTCGTACAGTCCTTCCCTGAAGGCTTTACATTTGAGCGGACATTGTCTTTTGAAGATGGTGCTATTGTCAAAGTAGAGTCTGATATTTCCATAGAGGACGGTAAGTTCGTTGGAAAAATTAAGTATAACGGAGAGGGCTTTCCAGAAGACGGCCCGGTTATGAAGAAAGAGGTCACTAAACTTGAACCTTCCTCAGAATCTATGTATGTCAGCGATGGCACCTTGGTAGGTGAAGTGGTTTTGTCATATAAGACCCAATCTACGCATTATACTTGCCATATGAAAACTATCTACCGCTCAAAAAAGCCAGTTGAGAATTTGCCAAAATTCCATTATGTCCATCACAGACTTGAGAAGAAAATCGTTGAAGAGGGGTATTATTACGAGCAACATGAAACCGCTATCGCCAAGCCTTGA |
| mCherry2 | ATGGTGAGCAAGGGCGAGGAGGATAACATGGCCATCATCAAGGAGTTCATGCGCTTCAAGGTGCACATGGAGGGCTCCGTGAACGGCCACGAGTTCGAGATCGAGGGCGAGGGCGAGGGCCGCCCCTACGAGGGCACCCAGACCGCCAAGCTGAAGGTGACCAAGGGTGGCCCCCTGCCCTTCGCCTGGGACATCCTGTCCCCTCAGTTCATGTACGGCTCCAAGGCCTACGTGAAGCACCCCGCCGACATCCCCGACTACTTGAAGCTGTCCTTCCCCGAGGGCTTCAATTGGGAGCGCGTGATGAACTTCGAGGACGGCGGCGTGGTGACCGTGACCCAGGACTCCTCCCTGCAGGACGGCGAGTTCATCTACAAGGTGAAGCTGCGCGGCACCAACTTCCCCTCCGACGGCCCCGTAATGCAGTGTCGTACCATGGGCTGGGAGGCCTCCACTGAGCGGATGTACCCCGAGGACGGCGCCCTGAAGGGCGAGATCAAGCAGAGGCTGAAGCTGAAGGACGGCGGCCACTACGACGCTGAGGTCAAGACCACCTACAAGGCCAAGAAGCCCGTGCAGCTGCCCGGCGCCTACAACGTCGACATCAAGTTGGACATCCTTTCCCACAACGAGGACTACACCATCGTGGAACAGTACGAACGCGCCGAGGGCCGCCACTCCACCGGCGGCATGGACGAGCTGTACAAG |
| H2B-CoGFPv0 | ATGCCAGAGCCAGCGAAGTCTGCTCCCGCCCCGAAAAAGGGCTCCAAGAAGGCGGTGACTAAGGCGCAGAAGAAAGGCGGCAAGAAGCGCAAGCGCAGCCGCAAGGAGAGCTATTCCATCTATGTGTACAAGGTTCTGAAGCAGGTCCACCCTGACACCGGCATTTCGTCCAAGGCCATGGGCATCATGAATTCGTTTGTGAACGACATTTTCGAGCGCATCGCAGGTGAGGCTTCCCGCCTGGCGCATTACAACAAGCGCTCGACCATCACCTCCAGGGAGATCCAGACGGCCGTGCGCCTGCTGCTGCCTGGGGAGTTGGCCAAGCACGCCGTGTCCGAGGGTACTAAGGCCATCACCAAGTACACCAGCGCTAAGATGTCCATTCCAGAGAACAGCGGCCTGACAGAGGAAATGCCGGCCCAGATGAACCTGGAGGGGGTCGTAAATGGCCATGCGTTTTCAATGGAAGGGATCGGGGGTGGAAACATACTGACTGGTATTCAAAAGTTGGACATTCGAGTGATTGAGGGTGACCCGCTCCCTTTCTCTTTTGACATCCTCTCCGTGGCATTTCAGTATGGTAATAGAACGTACACTTCCTATCCAGCAAAGATACCTGACTATTTCGTACAGTCCTTCCCTGAAGGCTTTACATTTGAGCGGACATTGTCTTTTGAAGATGGTGCTATTGTCAAAGTAGAGTCTGATATTTCCATAGAGGACGGTAAGTTCGTTGGAAAAATTAAGTATAACGGAGAGGGCTTTCCAGAAGACGGCCCGGTTATGAAGAAAGAGGTCACTAAACTTGAACCTTCCTCAGAATCTATGTATGTCAGCGATGGCACCTTGGTAGGTGAAGTGGTTTTGTCATATAAGACCCAATCTACGCATTATACTTGCCATATGAAAACTATCTACCGCTCAAAAAAGCCAGTTGAGAATTTGCCAAAATTCCATTATGTCCATCACAGACTTGAGAAGAAAATCGTTGAAGAGGGGTATTATTACGAGCAACATGAAACCGCTATCGCCAAGCCTTGA |
| mWasabi-ER | ATGCTGCTATCCGTGCCGTTGCTGCTCGGCCTCCTCGGCCTGGCCGTCGCCGACCGGTCGCACACCATGGTGAGCAAGGGCGAGGAGACCACAATGGGCGTAATCAAGCCCGACATGAAGATCAAGCTGAAGATGGAGGGCAACGTGAATGGCCACGCCTTCGTGATCGAGGGCGAGGGCGAGGGCAAGCCCTACGACGGCACCAACACCATCAACCTGGAGGTGAAGGAGGGAGCCCCCCTGCCCTTCTCCTACGACATTCTGACCACCGCGTTCAGTTACGGCAACAGGGCCTTCACCAAGTACCCCGACGACATCCCCAACTACTTCAAGCAGTCCTTCCCCGAGGGCTACTCTTGGGAGCGCACCATGACCTTCGAGGACAAGGGCATCGTGAAGGTGAAGTCCGACATCTCCATGGAGGAGGACTCCTTCATCTACGAGATACACCTCAAGGGCGAGAACTTCCCCCCCAACGGCCCCGTGATGCAGAAGGAGACCACCGGCTGGGACGCCTCCACCGAGAGGATGTACGTGCGCGACGGCGTGCTGAAGGGCGACGTCAAGATGAAGCTGCTGCTGGAGGGCGGCGGCCACCACCGCGTTGACTTCAAGACCATCTACAGGGCCAAGAAGGCGGTGAAGCTGCCCGACTATCACTTTGTGGACCACCGCATCGAGATCCTGAACCACGACAAGGACTACAACAAGGTGACCGTTTACGAGATCGCCGTGGCCCGCAACTCCACCGACGGCATGGACGAGCTGTACAAGAGATCGTACAAGAAGGACGAGCTG |
| mCherry2-ER | ATGCTGCTATCCGTGCCGTTGCTGCTCGGCCTCCTCGGCCTGGCCGTCGCCGACCGGTCGCACACCATGGTGAGCAAGGGCGAGGAGGATAACATGGCCATCATCAAGGAGTTCATGCGCTTCAAGGTGCACATGGAGGGCTCCGTGAACGGCCACGAGTTCGAGATCGAGGGCGAGGGCGAGGGCCGCCCCTACGAGGGCACCCAGACCGCCAAGCTGAAGGTGACCAAGGGTGGCCCCCTGCCCTTCGCCTGGGACATCCTGTCCCCTCAGTTCATGTACGGCTCCAAGGCCTACGTGAAGCACCCCGCCGACATCCCCGACTACTTGAAGCTGTCCTTCCCCGAGGGCTTCAATTGGGAGCGCGTGATGAACTTCGAGGACGGCGGCGTGGTGACCGTGACCCAGGACTCCTCCCTGCAGGACGGCGAGTTCATCTACAAGGTGAAGCTGCGCGGCACCAACTTCCCCTCCGACGGCCCCGTAATGCAGTGTCGTACCATGGGCTGGGAGGCCTCCACTGAGCGGATGTACCCCGAGGACGGCGCCCTGAAGGGCGAGATCAAGCAGAGGCTGAAGCTGAAGGACGGCGGCCACTACGACGCTGAGGTCAAGACCACCTACAAGGCCAAGAAGCCCGTGCAGCTGCCCGGCGCCTACAACGTCGACATCAAGTTGGACATCCTTTCCCACAACGAGGACTACACCATCGTGGAACAGTACGAACGCGCCGAGGGCCGCCACTCCACCGGCGGCATGGACGAGCTGTACAAGAGATCGTACAAGAAGGACGAGCTG |
| mCherry2-mito | ATGTCCGTCCTGACGCCGCTGCTGCTGCGGGGCTTGACAGGCTCGGCCCGGCGGCTCCCAGTGCCGCGCGCCAAGATCCATTCGTTGGGGGATCCACCGGTCGCCACCATGGTGAGCAAGGGCGAGGAGGATAACATGGCCATCATCAAGGAGTTCATGCGCTTCAAGGTGCACATGGAGGGCTCCGTGAACGGCCACGAGTTCGAGATCGAGGGCGAGGGCGAGGGCCGCCCCTACGAGGGCACCCAGACCGCCAAGCTGAAGGTGACCAAGGGTGGCCCCCTGCCCTTCGCCTGGGACATCCTGTCCCCTCAGTTCATGTACGGCTCCAAGGCCTACGTGAAGCACCCCGCCGACATCCCCGACTACTTGAAGCTGTCCTTCCCCGAGGGCTTCAATTGGGAGCGCGTGATGAACTTCGAGGACGGCGGCGTGGTGACCGTGACCCAGGACTCCTCCCTGCAGGACGGCGAGTTCATCTACAAGGTGAAGCTGCGCGGCACCAACTTCCCCTCCGACGGCCCCGTAATGCAGTGTCGTACCATGGGCTGGGAGGCCTCCACTGAGCGGATGTACCCCGAGGACGGCGCCCTGAAGGGCGAGATCAAGCAGAGGCTGAAGCTGAAGGACGGCGGCCACTACGACGCTGAGGTCAAGACCACCTACAAGGCCAAGAAGCCCGTGCAGCTGCCCGGCGCCTACAACGTCGACATCAAGTTGGACATCCTTTCCCACAACGAGGACTACACCATCGTGGAACAGTACGAACGCGCCGAGGGCCGCCACTCCACCGGCGGCATGGACGAGCTGTACAAG |
